# GADD45B Promotes Glucose-Induced Renal Tubular Epithelial-Mesenchymal Transition and Apoptosis *via* the p38 MAPK and JNK Signaling Pathways

**DOI:** 10.3389/fphys.2020.01074

**Published:** 2020-09-04

**Authors:** Mei Xue, Hongxi Sun, Rong Xu, Yue Wang, Jun Guo, Xiaoyu Li, Ying Cheng, Chaofei Xu, Chao Tang, Bei Sun, Liming Chen

**Affiliations:** NHC Key Laboratory of Hormones and Development, Tianjin Key Laboratory of Metabolic Diseases, Tianjin Medical University Chu Hsien-I Memorial Hospital and Tianjin Institute of Endocrinology, Tianjin Medical University, Tianjin, China

**Keywords:** GADD45B, p38 MAPK, JNK, diabetes, renal tubular injury

## Abstract

Growth arrest and DNA damage-inducible beta (GADD45B) is closely linked with cell cycle arrest, DNA repair, cell survival, or apoptosis in response to stress and is known to regulate the mitogen-activated protein kinase (MAPK) pathway. Here, using an RNA sequencing approach, we determined that GADD45B was significantly upregulated in diabetic kidneys, which was accompanied by renal tubular epithelial-mesenchymal transition (EMT) and apoptosis, as well as elevated MAPK pathway activation. *In vitro*, GADD45B expression in cultured human kidney proximal tubular epithelial cells (HK-2 cells) was also stimulated by high glucose (HG). In addition, overexpression of GADD45B in HK-2 cells exacerbated renal tubular EMT and apoptosis and increased p38 MAPK and c-Jun N-terminal kinases (JNK) activation, whereas knockdown of GADD45B reversed these changes. Notably, the activity of extracellular regulated kinase (ERK) was not affected by GADD45B expression. Furthermore, inhibitors of p38 MAPK (SB203580) and JNK (SP600125) alleviated HG‐ and GADD45B overexpression-induced renal tubular epithelial-mesenchymal transition and apoptosis. These findings indicate a role of GADD45B in diabetes-induced renal tubular EMT and apoptosis *via* the p38 MAPK and JNK pathways, which may be an important mechanism of diabetic kidney injury.

## Introduction

Diabetes mellitus has already become a common health problem worldwide. The latest data reported by the International Diabetes Federation show that in 2019, approximately 463 million adults were diagnosed with diabetes, and the global prevalence is predicted to increase to 700 million by 2045 (Saeedi et al., 2019). Poor glycemic control in patients with diabetes can cause serious microvascular complications, and diabetic kidney disease (DKD) is the leading cause of end-stage renal disease (Colhoun and Marcovecchio, 2018). DKD is clinically characterized by proteinuria and a progressive decrease in kidney function and is pathologically characterized by glomerular mesangial expansion, basement membrane thickening, podocyte injury, endothelial cell dysfunction, glomerulosclerosis, and tubulointerstitial fibrosis (Najafian et al., 2011; Vallon and Komers, 2011; Pourghasem et al., 2015).

Although current studies mainly focus on glomerular pathology, it has been well-documented that renal tubular epithelial lesions play an important role in the pathogenesis of DKD (Gilbert, 2017; Eriguchi et al., 2018). Renal epithelial-mesenchymal transition (EMT) is a pathological process that is observed in multiple kidney disease models (Chan et al., 2018; Xue et al., 2018). During the EMT process, epithelial cells gradually lose their features, including the downregulation of E-cadherin, while mesenchymal features are acquired, including the upregulation of Vimentin and α-smooth muscle actin (α-SMA; Carew et al., 2012). Type I EMT is necessary to promote embryonic development and organ formation, whereas type II EMT during renal tubular injury in DKD leads to tubulointerstitial fibrosis (Carew et al., 2012). In addition, apoptosis of tubular epithelial cells is clearly recognized as the pivotal pathogenesis of tubulointerstitial fibrosis. Accumulating evidence has shown that high glucose (HG) initiates oxidative stress or endoplasmic reticulum stress and subsequently promotes tubular epithelial cell apoptosis in DKD (Lau et al., 2012; Xiao et al., 2014; Huang et al., 2017). Growth arrest and DNA damage-inducible beta (GADD45B), a ubiquitously expressed protein, is a member of the GADD45 gene family that participates in mediating cell cycle arrest, DNA damage repair, and apoptosis in response to cell injury (Salvador et al., 2013). Abnormal expression of GADD45 has been indicated to be linked with a variety of diseases, such as tumors (Ju et al., 2009; Verzella et al., 2018) and nephropathy (Pippin et al., 2003). Studies have shown that GADD45B is considered a prognostic and predictive biomarker in colorectal cancer (Zhao et al., 2018) and that increased levels of GADD45B predict improved survival of prostate cancer patients (Huang et al., 2018). Moreover, gene profiling revealed the upregulation of GADD45B in glomeruli isolated from mice with podocyte-specific deletion of dicer (Shi et al., 2008). Recently, one research team found that the inhibition of GADD45B prevented proteinuria and podocyte injury in a glomerular disease model (Chen et al., 2016; Wang et al., 2018; Zhai et al., 2019).

Aberrant activation of mitogen-activated protein kinase (MAPK) caused by different stressful stimuli seems to be a key contributor to multiple cellular disorders, and three classic MAPK cascades include p38 kinase, c-Jun N-terminal kinases (JNK), and extracellular regulated kinase (ERK; Kim and Choi, 2015). Evidence suggests that the activated MAPK family may cause severe diabetic complications. For example, MAPK signaling is involved in regulating ischemia-reperfusion injury in diabetic hearts (Yu et al., 2018). p38 MAPK has been implicated as a critical factor in renal tubular epithelial cell apoptosis in db/db mice (Wu et al., 2015). Another study showed that dual inhibition of p38 and JNK activity impeded podocyte dysfunction and the progression of DKD (Denhez et al., 2019). Our research group found increased phosphorylation of ERK in kidneys from streptozotocin-induced diabetic rats and cultured podocytes exposed to HG (Chang et al., 2017). In addition, GADD45B was shown to modulate the MAPK cascade, thus further regulating podocyte apoptosis (Chen et al., 2016). However, whether MAPK signaling participates in GADD45B-mediated actions on renal tubular EMT, and apoptosis is poorly understood.

In the present study, we aimed to investigate the effect of GADD45B on renal tubular EMT and apoptosis in the context of diabetes and to further explore whether the MAPK pathway is involved in this regulation process.

## Materials and Methods

### Experimental Animals

Eight-week-old male db/db mice lacking the leptin receptor on a C57BL/KsJ background were used as spontaneous type 2 diabetes models. Wild-type littermate db/m mice were used as the nondiabetic controls. All experimental mice were purchased from the Model Animal Research Center of Nanjing University (Nanjing, China). The animals were housed in specific pathogen-free conditions with a light: dark cycle of 12:12 h, temperature of 22 ± 2°C, and humidity of 50 ± 3%. The mice had free access to water and were fed a standard diet for 8 weeks. This study was in compliance with the regulations and guidelines of the Animal Research Center and approved by the Ethics Committee of Tianjin Medical University (Tianjin, China).

The blood glucose levels of db/db mice all reached the diagnostic criteria for diabetes (≥16.7 mM), and those of db/m normal mice were between 6 and 12 mM. Each mouse was placed in an individual metabolic cage, and urine was collected for 24 h. At 16 weeks of age, the mice were anesthetized and sacrificed. After collecting blood samples, the left kidney weight (KW) was measured, and kidney tissues were harvested for subsequent experiments.

### Blood and Urine Chemistry

The levels of blood glucose, creatinine, blood urea nitrogen (BUN), triglyceride (TG), and total cholesterol (TC) were measured by an automatic biochemical analyzer (Roche, Germany) in the laboratory of Tianjin Medical University Chu Hsien-I Memorial Hospital. The urinary albumin concentration was measured by an ELISA kit (Mlbio, China) according to the manufacturer’s instructions, and urinary albumin excretion (UAE) was calculated as the total amount in 24 h.

### Cell Culture and Treatments

Human proximal tubular epithelial cells were acquired from the Chinese Academy of Sciences Cell Bank (Shanghai, China) and maintained in DMEM/F12 medium (HyClone, United States) containing 10% fetal bovine serum (Gibco, United States) in a humidified 5% CO_2_ atmosphere at 37°C. Human kidney proximal tubular epithelial cells (HK-2) cells were plated in six-well plates and stimulated with d-glucose at different concentrations when the cells reached 60–70% confluence. Mannitol was used for osmolality control.

With regard to GADD45B knockdown or overexpression, HK-2 cells were transfected with GADD45B small interfering RNA (siRNA; 100 pmol for six-well plates; GenePharma, China) or GADD45B plasmids (4 μg for six-well plates; GeneCopoeia, China) and their negative control using Lipofectamine 2000 reagent (Invitrogen, United States) as described previously. The siRNA sequences used for transfection are listed in **Supplementary Table S1**. After 6 h of transfection, the cells were cultured in fresh, normal glucose (NG), or HG medium for 48 h. Then, the cells were divided into four groups as follows: (1) NG group, (2) HG group, (3) HG + si-Con group, and (4) HG + si-GADD45B group or (1) NG group, (2) HG group, (3) NG + Vector group, and (4) NG + p-EZ-GADD45B group.

To determine the specific role of the MAPK pathway in GADD45B-mediated renal tubular EMT and apoptosis, we used inhibitors of p38 MAPK (SB203580) and JNK (SP600125; Selleck, United States) under HG or GADD45B overexpression conditions.

### RNA Sequencing and Real-Time PCR Analysis

Total RNA was extracted from renal tissues or HK-2 cells using TRIzol reagent (Invitrogen, United States). Genome-wide gene expression profiling of the kidney (*n* = 3 per group) was performed by Beijing Genomics Institute using the BGISEQ platform (Beijing Genomics institution, China). Kyoto encyclopedia of genes and genomes (KEGG) pathway analysis was performed to identify enriched pathways in the differentially expressed genes between groups. Reverse transcription (Thermo Scientific, United States) was performed to synthesize cDNA. Real-time PCR (RT-PCR) was performed with SYBR Green dye (Sangon Biotech, China) on a CFX96 Manager system (Bio-Rad, United States). The relative gene expression was calculated and normalized to the expression of β-actin by the 2^−ΔΔCT^ method. All primers used in this study were designed and acquired from Tsingke Biological Technology (Beijing, China), and the nucleotide sequences of all primers are shown in **Supplementary Table S2**.

### Protein Extraction and Western Blot Analysis

Proteins from renal tissues or HK-2 cells were extracted with RIPA buffer (Solarbio, China) containing a protease inhibitor cocktail, and the protein concentration was determined by a BCA protein assay kit (Solarbio, China). Then, 40 μg of protein was separated by SDS/PAGE, transferred to nitrocellulose filter membranes, and blocked with 5% nonfat milk for 1 h at room temperature, and then the membranes were incubated with the following primary antibodies at 4°C overnight: anti-GADD45B (Invitrogen, United States, catalog# PA5-43160), anti-E-Cadherin (Proteintech, China, catalog# 20874-1-AP), anti-Vimentin (Proteintech, China, catalog# 10366-1-AP) and anti-α-SMA (Proteintech, China, catalog# 14395-1-AP), anti-Bcl2 (CST, United States, catalog# 3498), anti-Bax (CST, United States, catalog# 2772), anti-cleaved Caspase 3 (CST, United States, catalog# 9654), anti-Phospho-p38 MAPK (Thr180/Tyr182; CST, United States, catalog# 4511), anti-p38 MAPK (CST, United States, catalog# 9212), anti-Phospho-JNK (Thr183/Tyr185; CST, United States, catalog# 9251), anti-JNK (CST, United States, catalog# 9592), anti-phospho-ERK (Thr202/Tyr204; CST, United States, catalog# 4370), anti-ERK (CST, United States, catalog# 4695), and anti-β-actin (Bioworld, United States, catalog# BS6007M). After the blots were washed, the secondary antibody (Sungene Biotech, China, anti-rabbit: catalog# LK2001, anti-mouse: catalog# LK2003) was added and incubated for 1 h at room temperature. The immunoblots were detected with an ECL kit (Advansta, United States). Band intensity was analyzed using ImageJ software and normalized to the expression of β-actin.

### Renal Histological Examination

Kidney tissues were fixed with 4% paraformaldehyde and embedded in paraffin. Four-micrometer-thick sections were cut and then stained with hematoxylin-eosin (HE), Masson and periodic acid-schiff (PAS) (Solarbio, China) according to the manufacturer’s protocols. For immunohistochemical staining, after deparaffinization, rehydration, and antigen repair, the kidney sections were treated with 3% H_2_O_2_ for 10 min, blocked with 1% bovine serum albumin (Sigma, United States) for 1 h, and then incubated with specific primary antibodies against GADD45B (1:100; Invitrogen, United States, catalog# PA5-43160), E-Cadherin (1:500; Proteintech, China, catalog# 20874-1-AP), Vimentin (1:500; Proteintech, China, catalog# 10366-1-AP), and α-SMA (1:200; Proteintech, China, catalog# 14395-1-AP) at 4°C overnight. After being washed, the sections were incubated with the secondary antibody for 1 h at 37°C. Finally, diaminobenzidine (Zsbio, China) was added to the slides, which were then counterstained with hematoxylin (Solarbio, China). The pathological changes in kidney tissue and protein expression were viewed under a light microscope with a digital camera (Olympus, Japan).

### Immunofluorescence Staining

HK-2 cells were cultured on coverslips in a 24-well plate (Corning, United States). After treatment, the cells were fixed with 4% paraformaldehyde for 30 min, permeabilized with 0.1% Triton X-100 (Solarbio, China) for 30 min, and blocked with 1% bovine serum albumin (Sigma, United States) for 1 h. Rabbit anti-GADD45B primary antibody (1:50; Invitrogen, United States, catalog# PA5-43160) was added and incubated at 4°C overnight, followed by TRITC-conjugated secondary antibodies (Zsbio, China). After that, the cell nucleus was stained with DAPI (Zsbio, China). Images were captured by a fluorescence microscope equipped with a digital camera (Olympus, Japan).

### Statistical Analysis

GraphPad Prism 7.0 software was used to perform the statistical analyses. All values are expressed as the mean ± SD. One-way ANOVA with Tukey’s test was utilized to determine significant differences among multiple groups, and unpaired Student’s *t*-tests were used to compare the differences between two groups. Statistical significance was considered at a value of *p* < 0.05.

## Results

### db/db Mice Developed Detectable Diabetic Kidney Disease

**Figure 1:**
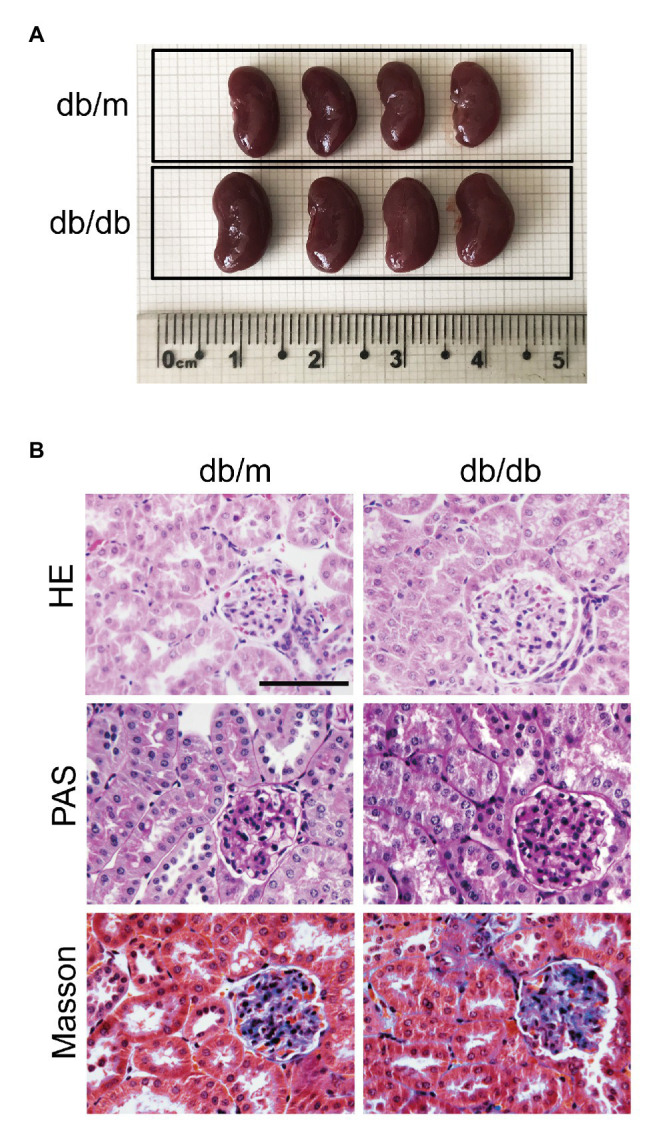
Renal histopathological changes in experimental mice at 16 weeks. **(A)** Representative images of whole kidneys from db/m and db/db mice. The scale bar is located on the image, and units are cm. **(B)** Representative images of kidney sections stained with HE, PAS, and Masson’s trichrome. Original magnification = 400. Scale bar = 100 μm.

Compared with those of the db/m mice, the body weight (BW), fasting blood glucose (FBG), TG, and TC levels of the db/db mice were significantly elevated (**Supplementary Table S3**). The kidney size in db/db mice was obviously larger, and the KW was evidently heavier than those in db/m mice, but the KW-to-BW ratio of db/db mice was reduced because of their increased BWs (**Figure 1A**, **Supplementary Table S3**). Moreover, increased levels of UAE, serum creatinine (Scr), and BUN were observed in diabetic mice (**Supplementary Table S3**). HE, Masson, and PAS staining was performed to examine the morphological changes in the kidney. Images of HE staining shown in **Figure 1** revealed glomerular enlargement, mesangial expansion, swollen, and dilated renal tubules, and decreased tubular lumen in db/db mice (**Figure 1B**). Masson and PAS staining also showed that increased glycogen was deposited in both the glomeruli and renal tubules, and glomerulosclerosis and tubulointerstitial fibrosis were discernible in db/db mice (**Figure 1B**). The UAE results, kidney function, and renal histopathological changes in db/db mice indicated that diabetic mice suffered severe renal impairment at 16 weeks of age, and we successfully established a DKD.

### GADD45B Was Upregulated in db/db Mice and HG-Induced HK-2 Cells

**Figure 2:**
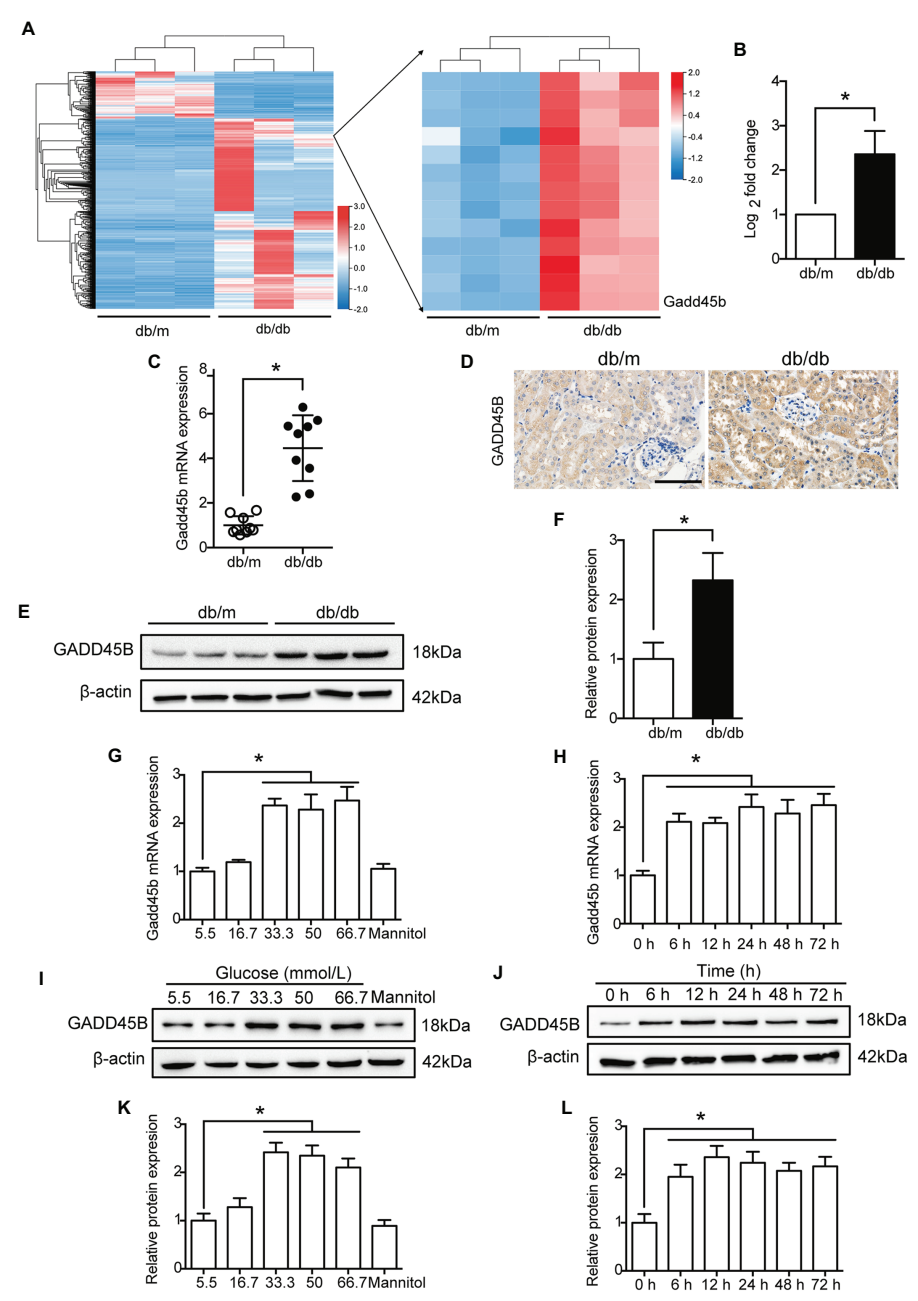
The expression of growth arrest and DNA damage-inducible beta (GADD45B) was increased in the kidneys of db/db mice and HG-induced HK-2 cells. **(A)** Heat map of the 1,194 differentially expressed genes (the logarithm of the fold change to base 2 > 2) and the right part is the enlarged view containing GADD45B. **(B)** The logarithm of the fold change to base 2 of GADD45B expression was determined by RNA sequencing analysis. **(C)** Real-time PCR (RT-PCR) analysis of GADD45B messenger RNA (mRNA) expression in kidney samples, *N* = 9. **(D)** Representative images of immunohistochemical staining of GADD45B in kidney sections. Original magnification = 400. Scale bar = 100 μm. **(E,F)** Western blot bands and quantitative analysis of GADD45B protein expression in kidney samples, *N* = 6. **(G,H)** RT-PCR analysis of GADD45B mRNA expression in HK-2 cells, *N* = 3. **(I–L)** Western blot bands and quantitative analysis of GADD45B protein expression in HK-2 cells, *N* = 3. The data are presented as the mean ± SD. ^*^ *p* < 0.05.

According to the RNA sequencing analysis the kidney, 3,642 genes were differentially expressed between the db/m and db/db groups. Among these genes, 2,748 showed increased expression and 894 showed decreased expression in diabetic kidneys compared to control kidneys, of which the logarithm of the fold change to base 2 of 1,194 genes was greater than 2 (**Supplementary Figure S1**, **Figure 2A**). The relative expression of GADD45B was higher in db/db mice than in db/m mice, and the logarithm of the fold change to base 2 was 2.364 (**Figure 2B**). Subsequently, we verified renal GADD45B expression by RT-PCR, immunohistochemical staining, and western blot. We found that GADD45B expression was apparently increased in the diabetic kidney compared with the db/m kidney (**Figures 2C**–**F**). More importantly, GADD45B was mainly distributed in the renal tubules, and glomerular GADD45B signals were relatively weak, as indicated by immunohistochemical staining (**Figure 2D**). The specific distribution of GADD45B motivated us to further perform an *in vitro* study in HK-2 cells. Similarly, the messenger RNA (mRNA) and protein levels of GADD45B in HK-2 cells were significantly increased not only under different glucose concentrations but also at different time points. Relative to the 5.5 mM group, 33.3, 50, or 66.7 mM glucose stimulated an apparent increase in GADD45B expression, while no significant differences were shown in the 16.7 mM group or mannitol control (**Figures 2G**,**I**,**K**). In addition, GADD45B expression in HK-2 cells was elevated after HG treatment for 6, 12, 24, 36, 48, or 72 h (**Figures 2H**,**J**,**L**). Therefore, glucose-stimulated GADD45B expression was not concentration-dependent or time-dependent in HK-2 cells.

### The Role of GADD45B in Controlling Renal Tubular EMT and Apoptosis *in vitro*

**Figure 3:**
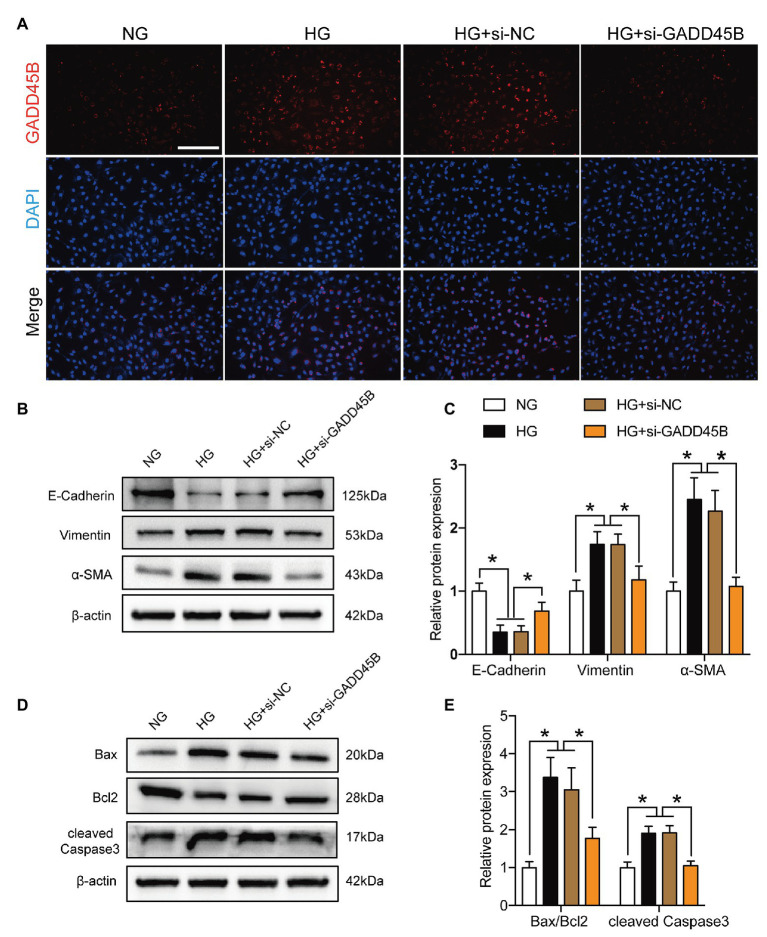
Effects of GADD45B knockdown on renal tubular epithelial-mesenchymal transition (EMT) and apoptosis in HK-2 cells under high glucose (HG) conditions. **(A)** Representative images of immunofluorescence staining of GADD45B in HK-2 cells. Original magnification = 200. Scale bar = 200 μm. **(B)** Western blot bands showing E-cadherin, Vimentin, and α-smooth muscle actin (α-SMA) protein expression in HK-2 cells. **(C)** Quantitative analysis of **(B)**, *N* = 3. **(D)** Western blot bands showing Bax, Bcl2, and cleaved Caspase 3 protein expression in HK-2 cells. **(E)** Quantitative analysis of **(D)**, *N* = 3. The data are presented as the mean ± SD. ^*^ *p* < 0.05.

**Figure 4:**
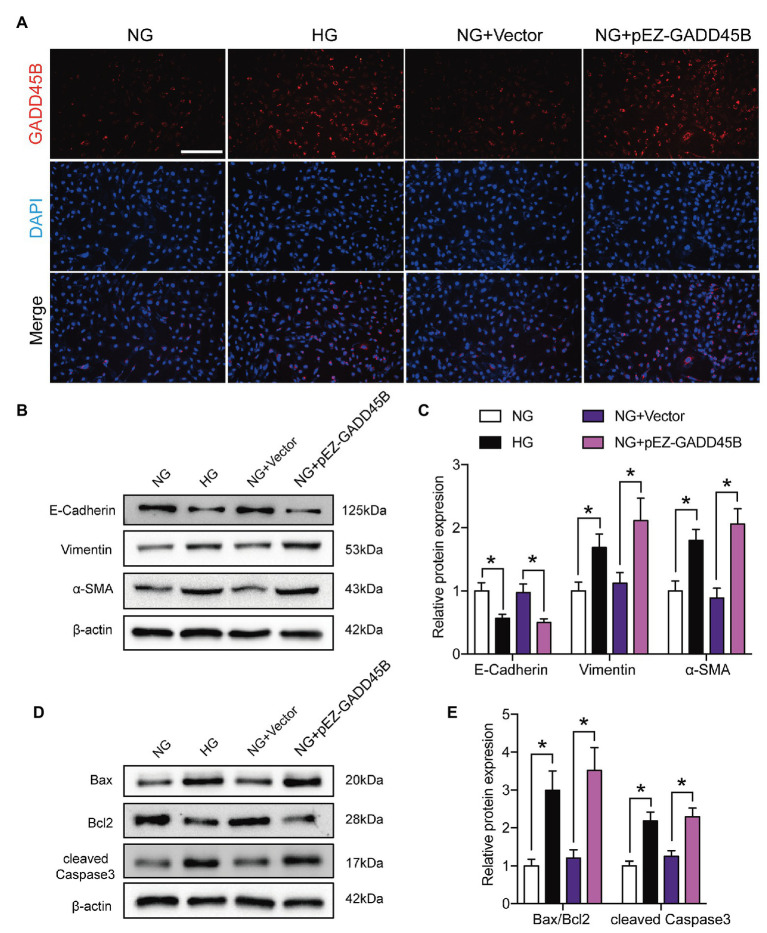
Effects of GADD45B overexpression on renal tubular EMT and apoptosis in HK-2 cells. **(A)** Representative images of immunofluorescence staining of GADD45B in HK-2 cells. Original magnification = 200. Scale bar = 200 μm. **(B)** Western blot bands showing E-cadherin, Vimentin, and α-SMA protein expression in HK-2 cells. **(C)** Quantitative analysis of **(B)**, *N* = 4. **(D)** Western blot bands showing Bax, Bcl2, and cleaved Caspase 3 protein expression in HK-2 cells. **(E)** Quantitative analysis of **(D),** *N* = 4. The data are presented as the mean ± SD. ^*^ *p* < 0.05.

Based on the above results, we hypothesized that the upregulation of GADD45B accounts for the proteinuria and renal tubular EMT and apoptosis observed in DKD. Therefore, we established GADD45B knockdown and overexpression models in HK-2 cells. Transfection of HK-2 cells with GADD45B siRNA or plasmids successfully led to the suppression or overexpression of GADD45B, respectively (**Supplementary Figures S2A–F**). Among the three siRNA sequences, GADD45B siRNA2 possessed the most obvious inhibitory effect at the mRNA and protein levels, and we used siRNA2 in all subsequent experiments (**Supplementary Figures S2A–C**). The reliable transfection effects were further verified by immunofluorescence staining (**Figures 3A**, **4A**). As shown in **Figures 3B**,**C**, after treatment with HG (33.3 mM) for 48 h, HK-2 cells exhibited EMT, as indicated by the decreased expression of E-Cadherin and increased expression of Vimentin and α-SMA. The same treatment also significantly increased the apoptosis level of HK-2 cells, as shown by the increased Bax-to-Bcl2 ratio and expression of cleaved Caspase 3 (**Figures 3D**,**E**). In contrast, inhibition of GADD45B largely rescued HK-2 cells from HG-induced EMT and apoptosis (**Figures 3B**–**E**). Furthermore, under NG conditions, overexpression of GADD45B also exacerbated the levels of EMT and apoptosis in HK-2 cells despite the lack of HG stimulation (**Figures 4B**–**E**). GADD45B overexpression had a similar effect as that of HG on HK-2 cell EMT and apoptosis. Thus, GADD45B is involved in maintaining HG-associated EMT and apoptosis in HK-2 cells.

### Effects of GADD45B Knockdown or Overexpression on the Activity of the MAPK Pathway

**Figure 5:**
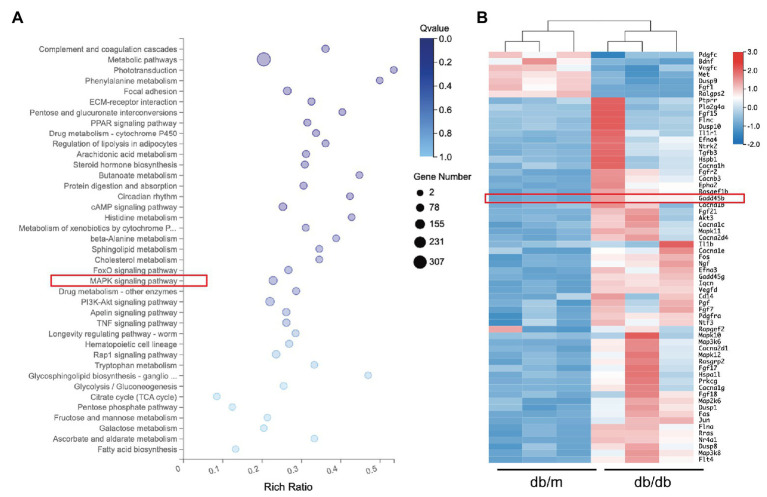
Results of RNA sequencing analysis. **(A)** Representative image of kyoto encyclopedia of genes and genomes (KEGG) analysis showing the enriched pathways in differentially expressed genes. **(B)** Heat map of the 63 mitogen-activated protein kinase (MAPK) pathway-related genes.

**Figure 6:**
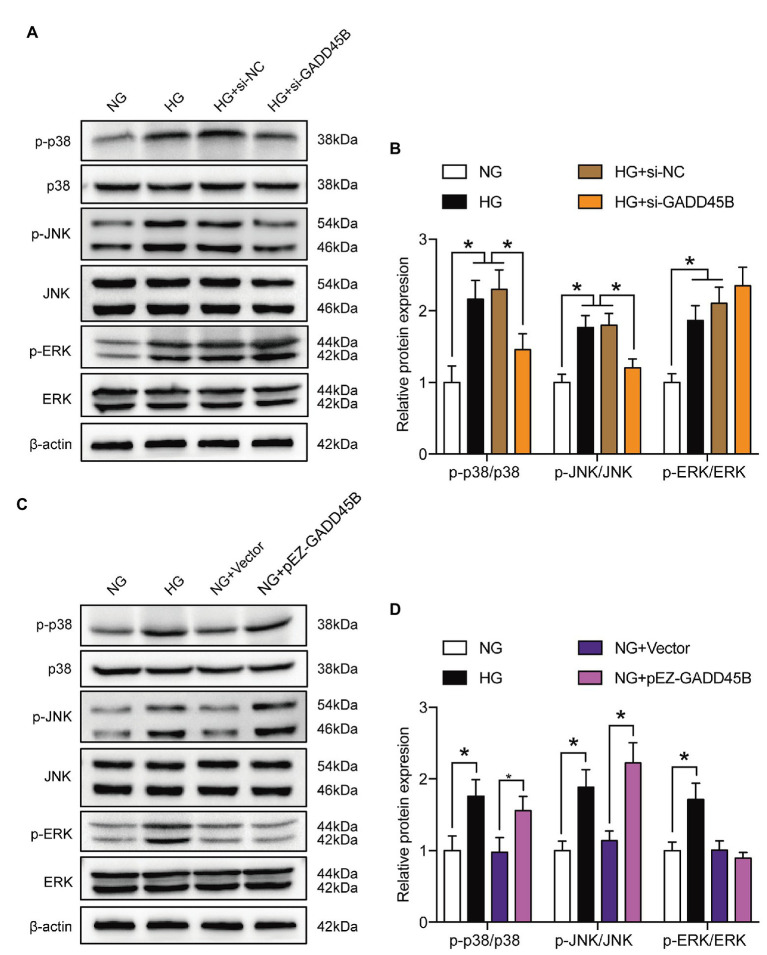
Effects of GADD45B on the activity of the MAPK signaling pathway in HK-2 cells. **(A,C)** Western blot bands showing p-p38 MAPK, p38 MAPK, p-c-Jun N-terminal kinase (JNK), JNK, p-extracellular regulated kinase (ERK), and ERK protein expression in HK-2 cells. **(B,D)** Quantitative analysis of **(A)** and **(C)**, *N* = 3. The data are presented as the mean ± SD. ^*^ *p* < 0.05.

KEGG pathway analysis from the RNA sequencing results revealed that 39 KEGG pathways were significantly identified (**Figure 5A**). Among the MAPK pathway, the expression of 63 relevant genes, including GADD45B was significantly altered (**Figure 5B**). To further elucidate whether the MAPK pathway is involved in mediating HG-induced EMT and apoptosis in HK-2 cells, we measured the levels of components of the classic MAPK signaling pathway, including p38 MAPK, JNK, and p44/42 MAPK (ERK1/2). The p-p38 MAPK/p38 MAPK, p-JNK/JNK, and p-ERK/ERK ratios were all relatively increased in HG-treated HK-2 cells compared with control cells, and GADD45B depletion significantly reduced the ratios of p-p38 MAPK/p38 MAPK and p-JNK/JNK but not p-ERK/ERK (**Figures 6A**,**B**). Furthermore, GADD45B overexpression imitated the excitatory effect of HG on p-p38 MAPK/p38 MAPK and p-JNK/JNK but did not affect p-ERK/ERK (**Figures 6C**,**D**). These findings suggest that the p38 MAPK and JNK pathways but not ERK play critical roles in regulating GADD45B-induced renal tubular EMT and apoptosis.

### Renal Tubular EMT, Apoptosis and Activation of the MAPK Pathway Were Increased in db/db Mice

**Figure 7:**
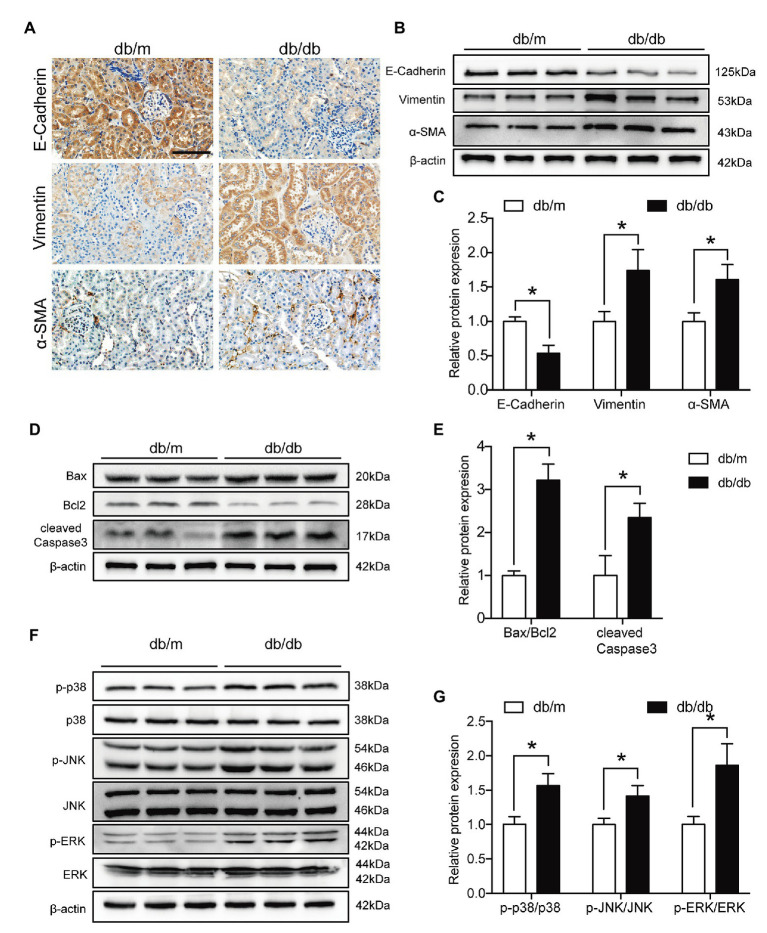
Renal tubular EMT and apoptosis and MAPK pathway activation were exacerbated in type 2 diabetic kidneys. **(A)** Representative images of immunohistochemical staining of E-cadherin, Vimentin, and α-SMA in kidney sections. Original magnification = 400. Scale bar = 100 μm. **(B)** Western blot bands showing E-cadherin, Vimentin, and α-SMA protein expression in the kidney samples. **(C)** Quantitative analysis of **(B)**, *N* = 9. **(D)** Western blot bands showing Bax, Bcl2, and cleaved Caspase 3 protein expression in the kidney samples. **(E)** Quantitative analysis of **(D)**, *N* = 9. **(F)** Western blot bands showing p-p38 MAPK, p38 MAPK, p-JNK, JNK, p-ERK, and ERK protein expression in the kidney samples. **(G)** Quantitative analysis of **(F)**, *N* = 9. The data are presented as the mean ± SD. ^*^ *p* < 0.05.

We next evaluated EMT levels *in vivo* by immunohistochemistry and observed that E-cadherin expression was markedly reduced in diabetic kidneys compared with db/m kidneys, but Vimentin and α-SMA expression levels were increased (**Figure 7A**). In addition, western blot analysis revealed that diabetes significantly exacerbated the levels of both renal EMT and apoptosis (**Figures 7B**–**E**). Moreover, the p-p38 MAPK/p38 MAPK, p-JNK/JNK, and p-ERK/ERK ratios were all increased in the kidneys of db/db mice (**Figures 7F**,**G**). These data suggested that renal tubular EMT, apoptosis, and activation of the MAPK pathway were increased both *in vivo* and *in vitro*.

### GADD45B Participates in HG-Induced Renal Tubular EMT and Apoptosis of HK-2 Cells *via* the p38 MAPK and JNK Signaling Pathways

**Figure 8:**
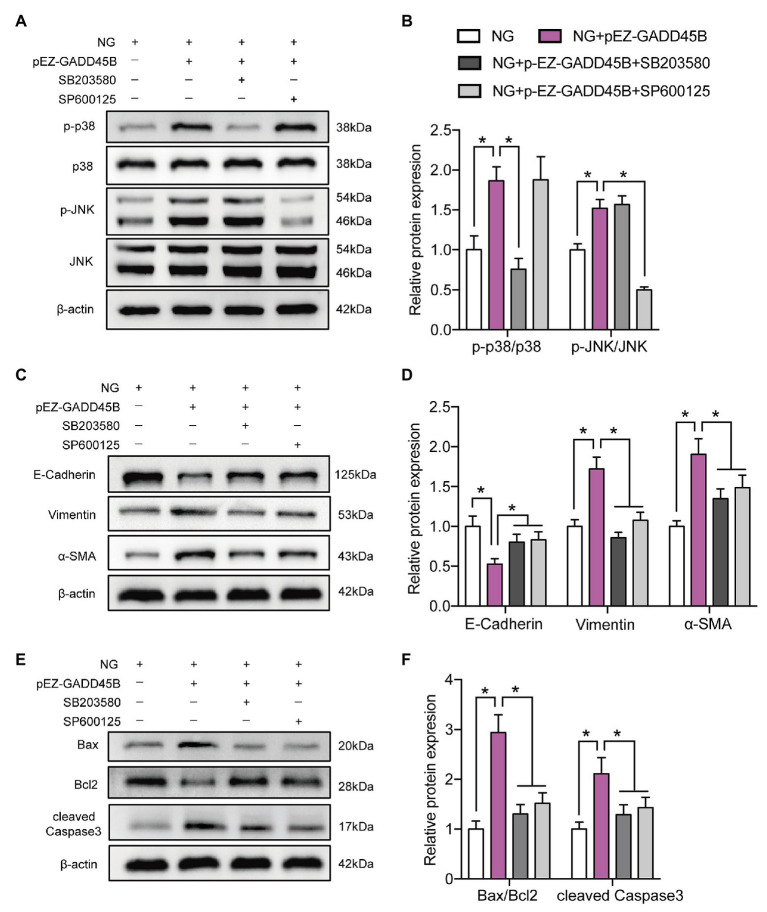
P38 MAPK and JNK inhibition attenuated GADD45B-induced renal tubular EMT and apoptosis. **(A)** Western blot bands showing p-p38 MAPK, p38 MAPK, p-JNK, and JNK protein expression in HK-2 cells. **(B)** Quantitative analysis of **(A)**, *N* = 3. **(C)** Western blot bands showing E-cadherin, Vimentin, and α-SMA protein expression in HK-2 cells. **(D)** Quantitative analysis of **(C)**, *N* = 3. **(E)** Western blot bands showing Bax, Bcl2, and cleaved Caspase 3 protein expression in HK-2 cells. **(F)** Quantitative analysis of **(E)**, *N* = 3. The data are presented as the mean ± SD. ^*^ *p* < 0.05.

To elucidate the exact mechanism by which GADD45B affects renal tubular injury through the p38 MAPK and JNK pathway signaling, we inactivated the probable pathways using specific inhibitors. As shown in **Figures 8A**,**B** and **Supplementary Figures S3A,B**, the p-p38 MAPK/p38 MAPK ratio was markedly decreased after treatment with SB203580 (inhibitor of p38 MAPK phosphorylation). SP600125 (inhibitor of JNK phosphorylation) treatment resulted in diminished expression of p-JNK/JNK. Due to the high selectivity of these inhibitors, SB203580 did not change the p-JNK/JNK ratio, and the p-p38 MAPK/p38 MAPK ratio was not affected by SP600125 in our study (**Figures 8A**,**B** and **Supplementary Figures S3A,B**). More importantly, both SB203580 and SP600125 significantly rescued HG‐ and GADD45B overexpression-induced EMT and apoptosis in HK-2 cells (**Figures 8C**–**F**, **Supplementary Figures S3C–F**). Hence, these results firmly confirmed that the p38 MAPK and JNK pathways participate in GADD45B-regulated renal tubular EMT and apoptosis.

## Discussion

Recent clinical and experimental evidence, as well as pathogenetic mechanisms, have supported a more central role of renal tubules in DKD (Zeni et al., 2017). In our study, we found that diabetic mice showed severe histopathological changes in renal tubules in DKD. To discover the key molecules and the critical pathways that regulate tubular injury, we measured the mRNA expression profile of mouse kidneys using RNA sequencing technology and subsequently quantified the expression of interesting target genes both *in vivo* and *in vitro*. Our data first showed an increase in GADD45B expression in diabetic kidneys and HK-2 cells exposed to HG. In response to environmental or physiological insults, the GADD45 protein is responsible for the coordination of cellular stress responses by interacting with a variety of partner proteins (Liebermann and Hoffman, 2008). For example, oxidative stress activates the GADD45B gene in the mouse liver (Kim et al., 2014), and GADD45A promotes UV-induced keratinocyte apoptosis (Hildesheim et al., 2002). Additionally, GADD45B mediates depressive-like behaviors induced by chronic social defeat stress through DNA demethylation (Labonte et al., 2019). However, whether GADD45B governs renal tubular injury in HK-2 cells remains unknown.

EMT has been linked to the progression of renal tubulointerstitial fibrosis (Das et al., 2019). Apoptosis also plays a critical role in renal tubular dysfunction, and studies have shown that apoptosis in renal tubular epithelial cells is a prominent factor associated with proteinuria in DKD (Habib, 2013). Our experiments have proven that both renal tubular EMT and apoptosis are exacerbated in type 2 diabetic mice and HG-stimulated HK-2 cells. Chen et al. (2016) observed that increased GADD45B expression was related to podocyte apoptosis. Similarly, it was shown that inhibition of GADD45B in HK-2 cells reduced HG-induced EMT and apoptosis, whereas GADD45B overexpression exacerbated EMT and apoptosis, which demonstrated that GADD45B is a key factor in regulating renal tubular EMT and apoptosis. However, it should be noted that GADD45B may have an antiapoptotic effect in other cell lines and different disease models. GADD45B may act as a protective effector to diminish ischemia-induced neuronal apoptosis and antimony-induced apoptosis in HEK293 cells (He et al., 2016; Jiang et al., 2016; Cho et al., 2019).

Next, we further explored the potential signaling pathway underlying the effects of GADD45B on renal tubular EMT and apoptosis. Accumulating evidence supports the increased activation of the MAPK signaling pathway in various human diseases (Kim and Choi, 2010). Moreover, it is widely known that the MAPK pathway serves as a common mediator of kidney injury (Cuarental et al., 2019), including DKD (Wu et al., 2015). Likewise, we observed that p38 MAPK, JNK, and ERK signaling were markedly upregulated in the diabetic kidney and HK-2 cells exposed to HG, which is consistent with the results of other studies (Wu et al., 2015; Chang et al., 2017; Denhez et al., 2019). Additionally, GADD45B ablation reduced the HG-induced activation of the p38 MAPK and JNK pathways, and GADD45B overexpression enhanced p38 MAPK and JNK pathway activation. Moreover, the inhibitory effects of a p38 inhibitor (SB203580) or JNK inhibitor (SP600125) diminished HG‐ or GADD45B overexpression-induced renal tubular EMT and apoptosis. These results established a definite relationship between GADD45B and renal tubular EMT and apoptosis in DKD through the activation of p38 MAPK and JNK.

It should be noted that the role of GADD45B in activating or inactivating the MAPK pathway and resulting in adverse or beneficial effects relies on the cell type and stressful conditions. The ERK pathway has been shown to have a key role in the development of DKD (Zhang et al., 2014; Chang et al., 2017), and our data indeed uncovered the activation of ERK both *in vivo* and *in vitro*, but altered GADD45B expression did not affect the activity of ERK in HK-2 cells, indicating that ERK is not a crucial signaling molecule in GADD45B-regulated renal tubular EMT and apoptosis. A previous study demonstrated that GADD45B overexpression resulted in the phosphorylation of p38 MAPK in podocytes but had no effect on the phosphorylation of JNK or ERK (Chen et al., 2016). In response to environmental stresses, however, GADD45B can activate the p38 and JNK pathways by binding and interacting with MAPK kinase kinase 4 (MEKK4) and subsequently mediating the activity of MAPK kinase 6 (MKK6; Takekawa and Saito, 1998). GADD45A could also lead to sustained p38 and JNK activity after UV radiation (Hildesheim et al., 2002), but GADD45A and GADD45B cooperated to protect hematopoietic cells from UV-induced apoptosis *via* p38 activation and JNK inhibition (Gupta et al., 2006). On the one hand, GADD45B decreased JNK phosphorylation by targeting MAPK kinase 4 (MKK4; Gupta et al., 2006; Kim et al., 2015). On the other hand, the physical interaction of GADD45B/MKK7 blunts the activity of MKK7, leading to the suppression of JNK activity (Papa et al., 2004, 2008). Pharmacologically disrupting the GADD45B/MKK7 complex could restore MKK7/JNK activation in multiple myeloma (Tornatore et al., 2014; Rega et al., 2018). Our data indicated that the p38 and JNK pathways, but not the ERK pathway, are activated by HG-mediated GADD45B overproduction in HK-2 cells. However, inadequate GADD45B may promote IL-1β-induced beta cell apoptosis due to a lack of inhibitory effects on JNK and ERK (Larsen et al., 2006). Therefore, a stable level of GADD45B should be maintained, and both increased and decreased expression will cause cell damage.

There are still some limitations in our study. First, RNA sequencing analysis showed the differential expression of a large number of MAPK pathway-related molecules in diabetic kidneys, and studies have illustrated that some of these molecules are involved in diabetic nephropathy. Denhez et al. (2019) recently confirmed that the deletion of a family of phosphatases responsible for MAPK inhibition (dual specificity phosphatases 4) promotes podocyte dysfunction and the progression of DKD. Therefore, other key molecules that can regulate diabetic renal tubular injury through the MAPK pathway need to be studied. Next, GADD45 family members are also involved in mediating other stress-related pathways, such as the p53 pathway (Hildesheim et al., 2002). It is unclear whether the action of GADD45B on renal tubular EMT and apoptosis in DKD occurs *via* these signaling pathways. Furthermore, we have not investigated the regulation of GADD45B on DKD at the animal level.

Taken together, our study showed that a high concentration of glucose stimulates GADD45B expression in the kidney tissue of mice and in cultured HK-2 cells. Aberrant GADD45B expression resulted in the activation of the p38 MAPK and JNK pathways, which contributed to renal tubular EMT and apoptosis. These data convincingly suggest that restraining renal GADD45B expression can provide a targeted approach to prevent renal tubular dysfunction and hinder the progression of DKD.

## Data Availability Statement

The raw data supporting the conclusions of this article will be made available by the authors, without undue reservation.

## Ethics Statement

The animal study was reviewed and approved by Ethical Committee of Tianjin Medical University.

## Author Contributions

LC and BS conceived and designed the research. MX and HS performed the experiments. RX and YW analyzed the data. JG and XL interpreted the results of the experiments. YC, CX, and CT prepared the figures. MX drafted the manuscript. HS edited and revised the manuscript. LC and BS approved the final version of the manuscript. All authors contributed to the article and approved the submitted version.

### Conflict of Interest

The authors declare that the research was conducted in the absence of any commercial or financial relationships that could be construed as a potential conflict of interest.

## Abbreviations

BUN: Blood urea nitrogen
BW: Body weight
DKD: Diabetic kidney disease
EMT: Epithelial-mesenchymal transition
ERK: Extracellular regulated kinase
FBG: Fasting blood glucose
GADD45B: Growth arrest and DNA damage-inducible beta
HG: High glucose
HK-2: Human kidney proximal tubular epithelial cells
JNK: c-Jun N-terminal kinases
KW: Kidney weight
MAPK: Mitogen-activated protein kinases
MKK: MAPK kinase
MEKK: MAPK kinase kinase
NG: Normal glucose
α-SMA: α-Smooth muscle actin
Scr: Serum creatinine
TC: Total cholesterol
TG: Triglyceride
UAE: Urinary albumin excretion.
