# Supplementary material for: GADD45B Promotes Glucose-Induced Renal Tubular Epithelial-Mesenchymal Transition and Apoptosis via the p38 MAPK and JNK Signaling Pathways
Source: Front Physiol. 2020 Sep 4;11:1074. doi: 10.3389/fphys.2020.01074 (PMC7508261; doi:10.3389/fphys.2020.01074)
Supplement: Supplementary file 1 [file Data_Sheet_1.doc]

**Supplemental table**

**Supplemental table 1. Primer sequences used for RT-PCR.**

| Gene | Primer sequences (5'-3') |
| --- | --- |
| Has GADD45B Forward | CCTGCAAATCCACTTCACGC |
| Has GADD45B Reverse | GTGTGAGGGTTCGTGACCAG |
| Has β-actin Forward | CTCACCATGGATGATGATATCGC |
| Has β-actin Reverse | CACATAGGAATCCTTCTGACCCA |
| Mus GADD45B Forward | GACATTGGGCACAACCGAAG |
| Mus GADD45B Reverse | GGATGAGGGGGCTGTCAAAT |
| Mus β-actin Forward | CCTCTATGCCAACACAGTGC |
| Mus β-actin Reverse | ACATCTGCTGGAAGGTGGAC |

**Supplemental table 2. Sequences of siRNA used for transfection.**

| Gene | Sequences (5'-3') |
| --- | --- |
| siRNA 1  GADD45B-Homo-360 | Sense CGGCCAAGUUGAUGAAUGUTT |
|  | Antisense ACAUUCAUCAACUUGGCCGTT |
| siRNA 2  GADD45B-Homo-480 | Sense GUGACAACGACAUCAACAUTT |
|  | Antisense AUGUUGAUGUCGUUGUCACTT |
| siRNA 3  GADD45B-Homo-649 | Sense GCCAGCUACUGCGAAGAAATT |
|  | Antisense UUUCUUCGCAGUAGCUGGCTT |
| Negative control | Sense UUCUCCGAACGUGUCACGUTT |
|  | Antisense ACGUGACACGUUCGGAGAATT |

**Supplemental table 3. Physical and biochemical values of db/m and db/db mice.**

|  | db/m | db/db |
| --- | --- | --- |
| BW（g） | 24.93±2.33 | 48.85±3.79* |
| FBG (mmol/L) | 9.06±1.43 | 29.28±3.07* |
| KW (mg) | 175±22.73 | 243±33.02* |
| KW/BW (mg/g) | 7.01±0.57 | 4.99±0.73 |
| UAE (μg/24h) | 11.77±5.08 | 172.85±23.07* |
| Scr (μmol/L) | 24.69±5.89 | 43.85±7.14* |
| BUN (mmol/L) | 5.38±1.07 | 9.52±1.22* |
| TG (U/L) | 0.62±0.10 | 1.72±0.27* |
| TC (U/L) | 2.01±0.52 | 4.52±0.71* |

BW, body weight; FBG, fasting blood glucose; KW, kidney weight; UAE, urinary albumin excretion; Scr, serum creatinine; BUN, blood urea nitrogen; TG, triglycerides; TC, total cholesterol. n = 10 per group. Data are expressed as mean ± SD. **P* < 0.05 vs. db/m group.

**Supplemental figure**

**Supplemental figure 1**

**
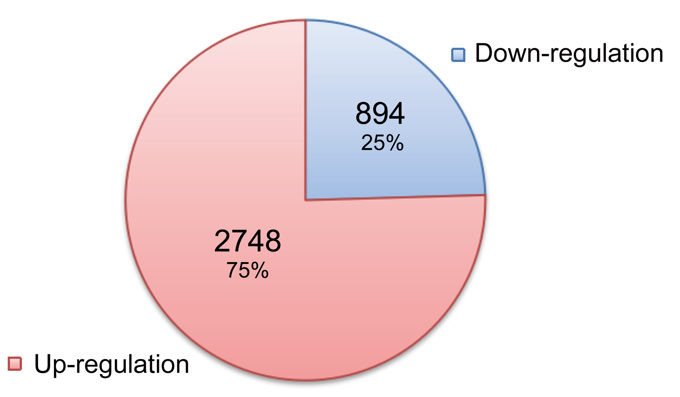
**

**Fig. S1. Result of RNA sequence analysis.** Number of up-regulated or down-regulated genes in db/db mice on comparison with db/m mice.

**Supplemental figure 2**

**
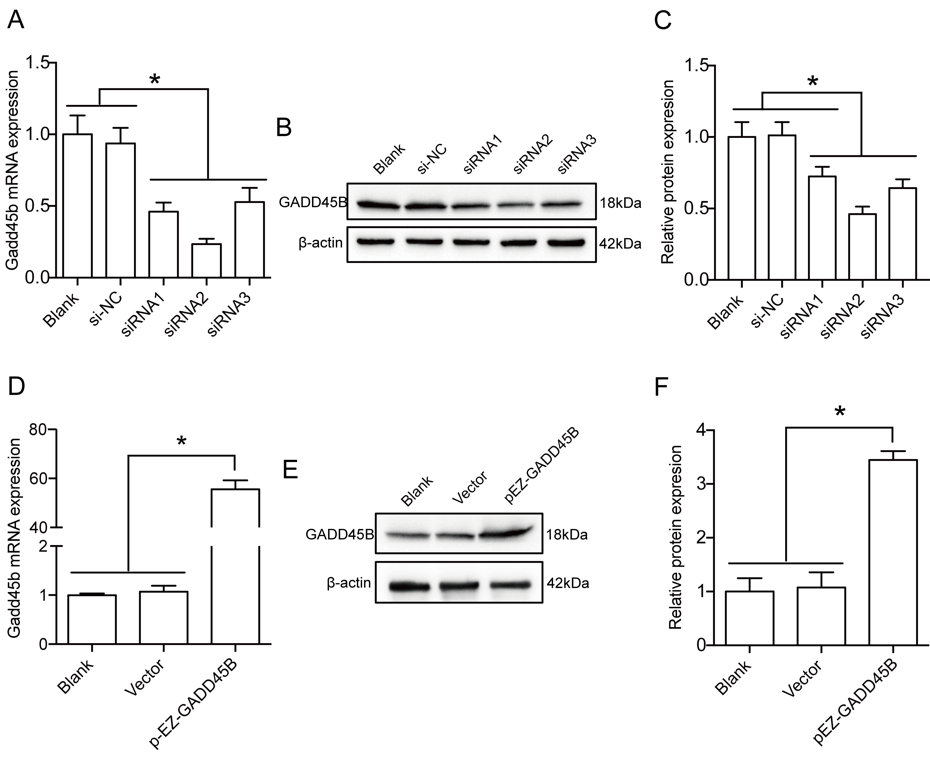
**

**Fig. S2. GADD45B expression levels of HK-2 cells after transfection.** (A) RT-PCR analysis of Gadd45b mRNA expression in HK-2 cells after transfected with siRNA. (B-C) Western blot bands and quantitative analysis of GADD45B protein expression in HK-2 cells after transfected with siRNA. (D) RT-PCR analysis of Gadd45b mRNA expression in HK-2 cells after transfected with plasmid. (E-F) Western blot bands and quantitative analysis of GADD45B protein expression in HK-2 cells after transfected with plasmid. Data are presented as mean±SD. **P*<0.05.

**Supplemental figure 3**

**
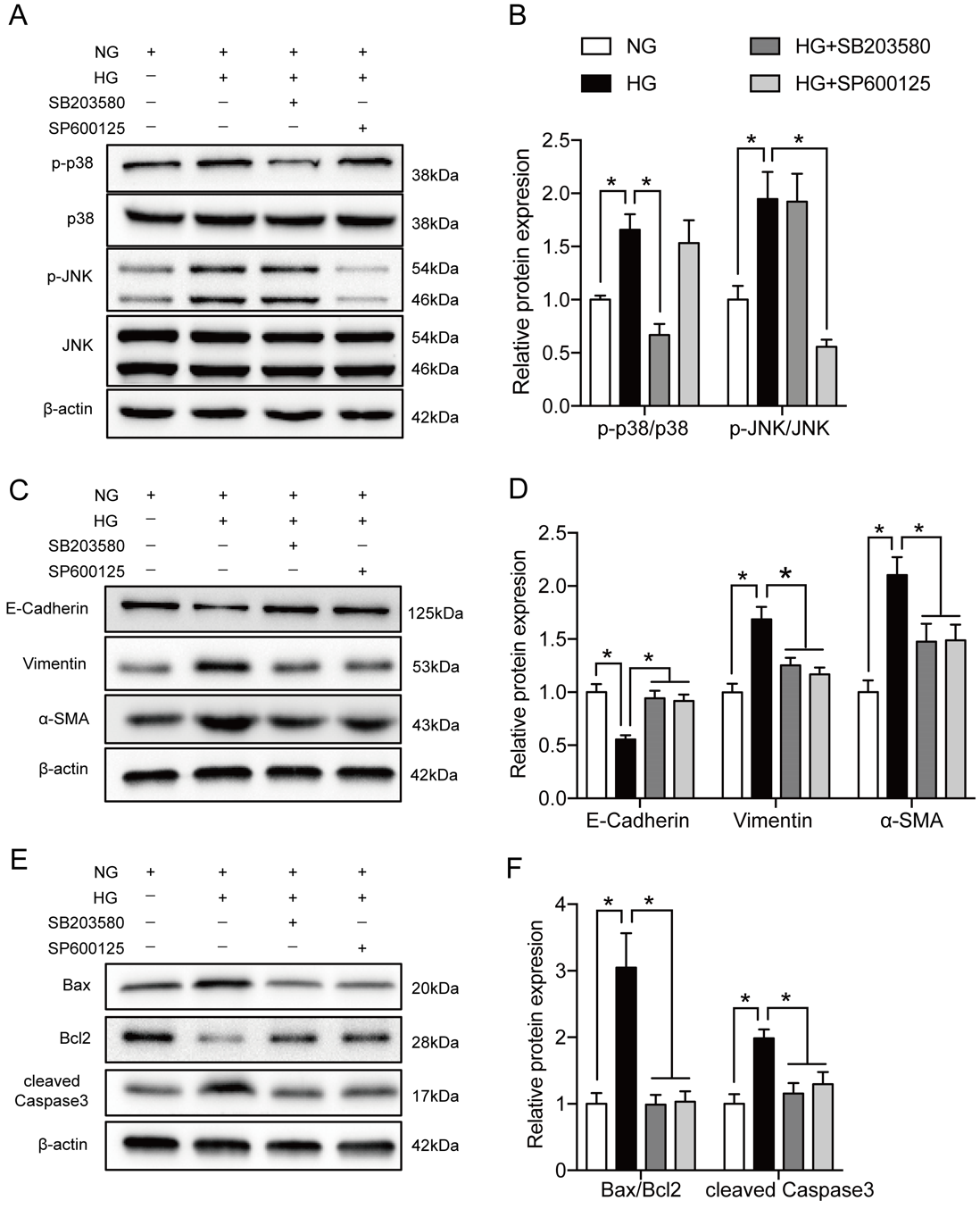
**

**Fig. S3. P38 MAPK and JNK inhibition reduced high glucose induced renal tubular injury.** (A) Western blot bands of p-p38 MAPK, p38 MAPK, p-JNK, and JNK protein expression in HK-2 cells. (B) Quantitative analysis of (A). (C) Western blot bands of E-cadherin, Vimentin, and α-SMA protein expression in HK-2 cells. (D) Quantitative analysis of (C). (E) Western blot bands of Bax, Bcl2, and cleaved Caspase3 protein expression in HK-2 cells. (F) Quantitative analysis of e. Data are presented as mean±SD. **P*<0.05.
